# Causal Relationship Between Inflammatory Bowel Disease and Neuropsychiatric Disorders: A Bidirectional Mendelian Randomization Study

**DOI:** 10.1002/brb3.71046

**Published:** 2025-11-11

**Authors:** Jiawei Lei, Liping Gong, Shan Wei, Junshan Deng

**Affiliations:** ^1^ Department of Gastrointestinal and Burn Surgery The First Affiliated Hospital of Hunan Traditional Chinese Medical College (Hunan Provincial Directly Affiliated Hospital of Traditional Chinese Medicine) Zhuzhou China

**Keywords:** inflammatory bowel disease, Mendelian randomization, neuropsychiatric disorders

## Abstract

**Background:**

Observational studies have indicated a correlation between inflammatory bowel disease (IBD) and neuropsychiatric disorders, yet the causal relationship remains unclear.

**Objective:**

We aim to employ two‐sample Mendelian randomization (MR) to ascertain the potential causal relationship between IBD and seven major neuropsychiatric disorders.

**Methods:**

We conducted a bidirectional two‐sample MR analysis utilizing genome‐wide association study (GWAS) summary statistics of European ancestry for IBD (31,665 cases and 33,977 controls) and its subtypes, ulcerative colitis (UC, 13,768 cases and 33,977 controls) and Crohn's disease (CD, 17,897 cases and 51,874 controls), along with seven major neuropsychiatric disorders: major depressive disorder (MDD, 135,458 cases and 344,901 controls), bipolar disorder (BD, 41,917 cases and 371,549 controls), schizophrenia (SCZ, 33,640 cases and 43,456 controls), Alzheimer's disease (AD, 39,106 cases and 46,828 controls), Parkinson's disease (PD, 33,674 cases and 449,056 controls), multiple sclerosis (MS, 14,498 cases and 24,091 controls), and amyotrophic lateral sclerosis (ALS, 27,205 cases and 110,881 controls).

**Results:**

Our analysis revealed potential positive causal effects of IBD and CD on MDD and SCZ. Similarly, SCZ was positively correlated with an increased risk of IBD and UC. There was a bidirectional positive association between IBD, UC, and MS, whereas CD showed a positive causal effect on MS. Similar to the investigation of the seven specified neuropsychiatric disorders on CD, we did not find evidence supporting causal effects of MDD, BD, AD, PD, or ALS on IBD or UC. Sensitivity analyses further reinforced the robustness of the MR estimates.

**Conclusion:**

Our results support potential causal relationships between IBD (including its subtypes CD and UC) and several neuropsychiatric disorders, reinforcing the gut‐brain axis concept and enhancing our understanding of extra‐intestinal manifestations of IBD and neuropsychiatric manifestations in the context of IBD.

## Graphical abstract

Forward represents the exploration of potential causal associations between inflammatory bowel disease (IBD)—including ulcerative colitis (UC) and Crohn's disease (CD)—and seven major neuropsychiatric disorders. Reverse indicates the opposite direction of investigation.

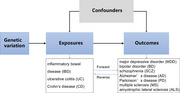

## 1 Introduction

Inflammatory bowel disease (IBD) encompasses a range of chronic, recurrent conditions characterized by inflammation of the gastrointestinal tract, affecting more than 2.5 million individuals across Europe (Molodecky et al. 2012). The two main types of IBD are ulcerative colitis (UC) and Crohn's disease (CD). IBD is believed to arise from a combination of genetic susceptibility and an abnormal immune response, leading to a persistent pro‐inflammatory state in the gut (Liu et al. 2015, Hedin et al. 2019). While the inflammation is most prominent within the gastrointestinal tract, there is increasing recognition of the extraintestinal manifestations of IBD, particularly its connection to neuropsychiatric symptoms (Ferro and Oliveira Santos 2021). A growing body of evidence highlights the importance of the gut‐brain axis, a complex bidirectional communication network that links the gastrointestinal system with the central nervous system (CNS). This pathway is increasingly regarded as a crucial mediator connecting gastrointestinal inflammation with CNS function and dysfunction (Gracie et al. 2019).

Neuropsychiatric symptoms, including mood disturbances, anxiety, and cognitive impairments, are notably more common in individuals with IBD compared to the general population. Reported prevalence rates of these symptoms in IBD patients range from 0.25% to 47.5%, with considerable variability depending on the study population and diagnostic methods (Ferro and Oliveira Santos 2021). Notably, studies have explored the bidirectional relationship between IBD and various neuropsychiatric conditions. For instance, research by Bisgaard has suggested a potential two‐way interaction between IBD and depression, where both the presence of IBD and depressive symptoms may influence each other (Bisgaard et al. 2022). Similarly, evidence supports a bidirectional association between IBD and multiple sclerosis (MS), suggesting that inflammatory processes in the gut may contribute to the development or exacerbation of MS, and vice versa (Kosmidou et al. 2017). There is also some indication that IBD may increase the risk of developing Parkinson's disease (PD), though the mechanisms behind this association remain unclear (Zhu et al. 2019).

Despite these findings, the degree of association between IBD and neuropsychiatric disorders remains inconsistent across studies, with significant variations in the reported strength and direction of these relationships (Fairbrass et al. 2022, Barberio et al. 2021). One of the key challenges in understanding this complex interaction is the unclear directionality of the association. While it is plausible that active IBD could lead to or exacerbate neuropsychiatric symptoms, it is equally possible that pre‐existing neuropsychiatric conditions may trigger or worsen IBD symptoms. This bidirectional relationship underscores the need for further research to unravel the complex interplay between IBD and neuropsychiatric health, potentially leading to more targeted treatment strategies that address both the gastrointestinal and psychological aspects of these diseases (Bonaz and Bernstein 2013).

Mendelian randomization (MR) is an epidemiological approach that utilizes genome‐wide association study (GWAS) data to infer causal relationships between exposure factors and outcomes (Skrivankova et al. 2021). MR is becoming a significant driving force in the field of neuropsychiatric diseases (Wang et al. 2022, Sun et al. 2023, Sun et al. 2024, Sun et al. 2024, Ma et al. 2024). Since genetic variants are randomly assigned at birth and do not change with disease occurrence and progression, the MR approach effectively overcomes the inherent limitations of observational studies (reverse causation, confounding factors) (Yuan and Larsson 2022).

Here, we aim to employ MR analysis to comprehensively assess the potential causal relationships between IBD (including UC and CD) and seven major neuropsychiatric disorders: major depressive disorder (MDD), bipolar disorder (BD), schizophrenia (SCZ), Alzheimer's disease (AD), PD, MS, and amyotrophic lateral sclerosis (ALS), thereby enhancing our understanding of the pathophysiology of the gut‐brain axis and informing related therapeutic strategies.

## 2 Methods

### 2.1 Study Design

**FIGURE 1:**
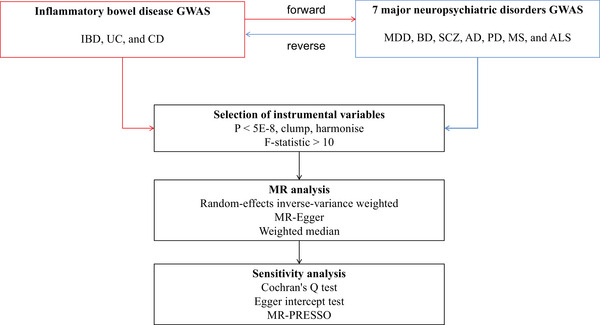
Research flowchart. **Abbreviations**: AD, Alzheimer's disease; ALS, amyotrophic lateral sclerosis; BD, bipolar disorder; CD, Crohn's disease; GWAS, genome‐wide association study; IBD, inflammatory bowel disease; MDD, major depressive disorder; MR, Mendelian randomization; MS, multiple sclerosis; PD, Parkinson's disease; SCZ, schizophrenia; UC, ulcerative colitis.

The workflow of this study is outlined in Figure 1. We systematically assessed the bidirectional causal relationships between IBD and seven major neuropsychiatric disorders using a two‐sample MR approach. To mitigate potential population stratification bias, we focused the MR analyses on European populations. Our study adheres to three core MR assumptions: the relevance assumption, which asserts a strong association between genetic instruments and the exposure; the independence assumption, which ensures that genetic instruments are not linked to confounders; and the exclusion restriction assumption, which states that genetic instruments affect the outcome solely through the exposure, not via alternative pathways (Burgess et al. 2018). All analyses were performed using the TwoSampleMR package in R software, a widely recognized tool for performing robust MR analyses with summary‐level GWAS data (Holmes et al. 2021).

### 2.2 Data Sources

#### 2.2.1 IBD, UC, and CD

The summary‐level data for IBD were sourced from the International IBD Genetics Consortium, which includes 31,665 patients and 33,977 controls of European ancestry. This dataset offers valuable biological insights into the pathogenesis of IBD, enabling a deeper understanding of its genetic underpinnings (Liu et al. 2015). Briefly, this study integrated genome‐wide datasets from 15 countries across Europe, North America, and Oceania, including eight UC cohorts and seven CD cohorts. The number of SNPs ranged from 124,888 to 157,116. The IBD cases comprised UC (13,768 patients and 33,977 controls) and CD (17,897 patients and 51,874 controls). All cases across cohorts met the clinical criteria for IBD. The diagnoses of IBD, including UC and CD, were determined through currently recognized radiological, endoscopic, and histopathological evaluations (Liu et al. 2015).

### 2.3 Seven Major Neuropsychiatric Disorders

Summary data for MDD were obtained from the Psychiatric Genomics Consortium (PGC), including 135,458 patients and 344,901 controls (Wray et al. 2018). MDD diagnoses were primarily determined through structured diagnostic interviews, national inpatient electronic records, self‐reported symptoms or treatments of MDD, or clinical diagnoses and treatments of depression reported by healthcare professionals (Wray et al. 2018). The GWAS meta‐analysis for BD included 41,917 patients and 371,549 controls from 57 cohorts of European ancestry (Mullins et al. 2021). This study identified 64 BD‐related loci, primarily enriched in synaptic signaling pathways and brain‐expressed genes (Mullins et al. 2021). Summary‐level data for SCZ came from the Schizophrenia Working Group of the Psychiatric Genomics Consortium, including 33,640 patients and 43,456 controls, identifying 128 significant loci (2014). AD summary data comprised 39,106 patients and 46,828 controls, revealing 75 risk loci, with pathway enrichment analysis highlighting the involvement of amyloid/tau pathways (Bellenguez et al. 2022). The summary data for PD were obtained from the International Parkinson's Disease Genomics Consortium, comprising 33,674 patients and 449,056 controls (Hemani et al. 2018). The summary data for MS were sourced from the International Multiple Sclerosis Genetics Consortium, which includes 14,498 cases and 24,091 controls (Beecham et al. 2013). This study employed the ImmunoChip custom genotyping array, identifying 48 MS susceptibility loci. The summary data for ALS were derived from the study by van Rheenen et al., which included 27,205 patients and 110,881 controls (2021). This investigation identified 15 risk loci, revealing the role of vesicular transport and autophagy in ALS. ALS diagnoses were made by neurologists specializing in motor neuron diseases based on the revised El Escorial Criteria (Brooks et al. 2000).

### 2.4 Selection of Instrumental Variables

Here, we employed a series of rigorous steps to obtain qualified instrumental variables. First, to obtain strongly associated genetic instruments, we set the significance threshold to *p* < 5E‐8 in the exposure GWAS summary statistics. Second, we performed clumping using the default parameters, with the European 1000 Genomes Project Phase 3 as the reference cohort, to minimize the effects of linkage disequilibrium. Third, we extracted the relevant parameters for the instrumental variables from the outcome data and used the harmonise_data function to ensure alignment between the exposure and outcome instruments. Fourth, we calculated the F‐statistic using *F* = BETA^2^/SE^2^, with an F‐statistic greater than 10 considered sufficient to reduce bias from weak instruments (Harroud et al. 2021, Burgess and Thompson 2011).

### 2.5 MR Analysis and Sensitivity Analysis

In the main analysis, we employed the Wald ratio method as the principal approach for examining causal relationships using a single instrumental variable. For analyses involving multiple instrumental variables, we utilized the random‐effects inverse‐variance weighted (IVW) method to assess the causal links between IBD and seven major neuropsychiatric disorders (Burgess et al. 2013). The IVW method assumes the validity of all included genetic variants and aggregates the Wald ratios from each genetic instrument to estimate the exposure‐outcome relationship. When horizontal pleiotropy is absent, IVW provides unbiased estimates, and its random‐effects variant accounts for potential heterogeneity among instruments (Burgess et al. 2017, Cai et al. 2022, Burgess et al. 2016). To further validate the robustness of our findings, we employed the MR‐Egger and weighted median methods as complementary approaches. These methods are designed to address potential biases arising from invalid instruments (Bowden et al. 2015, Bowden et al. 2016, Choi et al. 2019). Cochran's *Q* test was applied to assess the degree of heterogeneity within the instrumental variables, with a significant result indicating variability across the instruments (Haycock et al. 2016). To test for horizontal pleiotropy, an Egger intercept test was conducted, with a significant intercept suggesting the presence of pleiotropic effects (Bowden et al. 2015, Verbanck et al. 2018). In cases where horizontal pleiotropy was detected, the MR‐PRESSO method was applied to identify outliers and correct for pleiotropic bias, ensuring more accurate causal estimates (Verbanck et al. 2018). All analyses utilized the Genome Reference Consortium Human Build 37. Given that this is an exploratory analysis, no multiple testing corrections were applied to the *p*‐values.

## 3 Results

### 3.1 MR Analysis

#### 3.1.1 Forward Analysis

**FIGURE 2:**
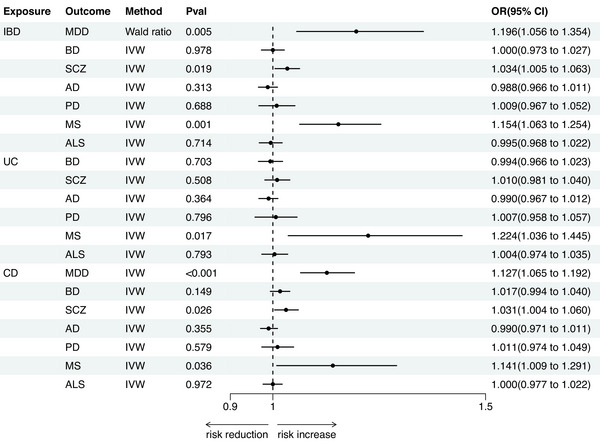
MR estimates of IBD on seven major neuropsychiatric disorders. **Abbreviations**: AD, Alzheimer's disease; ALS, amyotrophic lateral sclerosis; BD, bipolar disorder; CD, Crohn's disease; IBD, inflammatory bowel disease; MDD, major depressive disorder; MR, Mendelian randomization; MS, multiple sclerosis; PD, Parkinson's disease; SCZ, schizophrenia; UC, ulcerative colitis.

In all MR estimates, the F‐statistics for all genetic instruments were greater than 10, indicating no weak instrument bias. Notably, the analysis of UC and MDD was excluded due to insufficient genetic instruments. In the forward analysis, the IVW/Wald ratio method identified several significant causal associations between IBD and seven neuropsychiatric disorders (as shown in Figure 2, Table 1). Specifically, evidence was provided supporting a gene proxy for IBD being associated with an increased risk of three neuropsychiatric disorders: MDD (OR = 1.196, 95% CI = 1.056–1.354, *p* = 0.005), SCZ (OR = 1.034, 95% CI = 1.005–1.063, *p* = 0.019), and MS (OR = 1.154, 95% CI = 1.063–1.254, *p* = 0.001). Gene proxies for UC also exhibited a similar positive causal association with MS (OR = 1.224, 95% CI = 1.036–1.445, *p* = 0.017). CD was associated with an increased risk of three neuropsychiatric disorders: MDD (OR = 1.127, 95% CI = 1.065–1.192, *p* < 0.001), SCZ (OR = 1.031, 95% CI = 1.004–1.060, *p* = 0.026), and MS (OR = 1.141, 95% CI = 1.009–1.291, *p* = 0.036).

**TABLE 1:** MR‐Egger and weighted median analyses and sensitivity analysis results.

**Exposure**	**Outcome**	**Method**	Pval	OR	or_lci95	or_uci95	Cochran's Q test Pval	Egger intercept test Pval
Inflammatory bowel disease	Multiple sclerosis	MR‐Egger	0.648	1.046	0.862	1.271	<0.001	0.277
Inflammatory bowel disease	Multiple sclerosis	Weighted median	0.001	1.100	1.038	1.166		
Inflammatory bowel disease	Major depressive disorder	Wald ratio	0.005	1.196	1.056	1.354		
Inflammatory bowel disease	Bipolar disorder	MR‐Egger	0.051	0.938	0.881	1.000	<0.001	0.033
Inflammatory bowel disease	Bipolar disorder	Weighted median	0.380	0.986	0.957	1.017		
Inflammatory bowel disease	Schizophrenia	MR‐Egger	0.904	0.996	0.932	1.064	<0.001	0.223
Inflammatory bowel disease	Schizophrenia	Weighted median	0.894	0.998	0.964	1.032		
Inflammatory bowel disease	Alzheimer's disease	MR‐Egger	0.755	0.992	0.940	1.046	<0.001	0.896
Inflammatory bowel disease	Alzheimer's disease	Weighted median	0.400	0.989	0.964	1.015		
Inflammatory bowel disease	Parkinson's disease	MR‐Egger	0.299	1.054	0.955	1.163	<0.001	0.337
Inflammatory bowel disease	Parkinson's disease	Weighted median	0.532	1.018	0.962	1.077		
Inflammatory bowel disease	Amyotrophic lateral sclerosis	MR‐Egger	0.335	1.033	0.968	1.102	<0.001	0.22
Inflammatory bowel disease	Amyotrophic lateral sclerosis	Weighted median	0.268	0.981	0.948	1.015		
Ulcerative colitis	Multiple sclerosis	MR‐Egger	0.017	1.656	1.104	2.482	<0.001	0.113
Ulcerative colitis	Multiple sclerosis	Weighted median	0.001	1.111	1.046	1.179		
Ulcerative colitis	Bipolar disorder	MR‐Egger	0.637	0.983	0.917	1.054	<0.001	0.729
Ulcerative colitis	Bipolar disorder	Weighted median	0.624	1.008	0.976	1.041		
Ulcerative colitis	Schizophrenia	MR‐Egger	0.220	1.045	0.975	1.120	<0.001	0.294
Ulcerative colitis	Schizophrenia	Weighted median	0.754	0.994	0.958	1.032		
Ulcerative colitis	Alzheimer's disease	MR‐Egger	0.537	0.983	0.931	1.038	<0.001	0.789
Ulcerative colitis	Alzheimer's disease	Weighted median	0.287	0.985	0.958	1.013		
Ulcerative colitis	Parkinson's disease	MR‐Egger	0.697	0.977	0.867	1.100	<0.001	0.586
Ulcerative colitis	Parkinson's disease	Weighted median	0.817	1.007	0.950	1.068		
Ulcerative colitis	Amyotrophic lateral sclerosis	MR‐Egger	0.013	1.097	1.021	1.179	<0.001	0.01
Ulcerative colitis	Amyotrophic lateral sclerosis	Weighted median	0.783	0.995	0.959	1.032		
Crohn's disease	Multiple sclerosis	MR‐Egger	0.450	0.881	0.636	1.222	<0.001	0.097
Crohn's disease	Multiple sclerosis	Weighted median	0.000	1.121	1.066	1.178		
Crohn's disease	Bipolar disorder	MR‐Egger	0.071	0.947	0.893	1.004	<0.001	0.012
Crohn's disease	Bipolar disorder	Weighted median	0.842	0.998	0.973	1.022		
Crohn's disease	Schizophrenia	MR‐Egger	0.972	1.001	0.931	1.077	<0.001	0.395
Crohn's disease	Schizophrenia	Weighted median	0.255	1.017	0.988	1.048		
Crohn's disease	Alzheimer's disease	MR‐Egger	0.294	0.971	0.920	1.025	<0.001	0.446
Crohn's disease	Alzheimer's disease	Weighted median	0.197	0.985	0.963	1.008		
Crohn's disease	Parkinson's disease	MR‐Egger	0.151	1.077	0.974	1.191	<0.001	0.184
Crohn's disease	Parkinson's disease	Weighted median	0.661	1.011	0.962	1.062		
Crohn's disease	Amyotrophic lateral sclerosis	MR‐Egger	0.053	1.063	1.000	1.130	<0.001	0.037
Crohn's disease	Amyotrophic lateral sclerosis	Weighted median	0.076	1.027	0.997	1.057		
Multiple sclerosis	Inflammatory bowel disease	MR Egger	0.180	1.096	0.961	1.250	<0.001	0.797
Multiple sclerosis	Inflammatory bowel disease	Weighted median	0.000	1.125	1.088	1.164		
Major depressive disorder	Inflammatory bowel disease	MR Egger	0.780	6.963	0.000	1.08E+06	<0.001	0.763
Major depressive disorder	Inflammatory bowel disease	Weighted median	0.166	1.439	0.859	2.409		
Bipolar disorder	Inflammatory bowel disease	MR Egger	0.193	0.346	0.082	1.466	<0.001	0.145
Bipolar disorder	Inflammatory bowel disease	Weighted median	0.236	1.105	0.937	1.304		
schizophrenia	Inflammatory bowel disease	MR Egger	0.802	1.052	0.711	1.557	0.115	0.865
schizophrenia	Inflammatory bowel disease	Weighted median	0.061	1.083	0.996	1.177		
Alzheimer's disease	Inflammatory bowel disease	MR Egger	0.155	1.569	0.912	2.698	0.002	0.187
Alzheimer's disease	Inflammatory bowel disease	Weighted median	0.099	1.126	0.978	1.296		
Parkinson's disease	Inflammatory bowel disease	MR Egger	0.583	1.064	0.863	1.311	0.811	0.945
Parkinson's disease	Inflammatory bowel disease	Weighted median	0.012	1.093	1.019	1.173		
Amyotrophic lateral sclerosis	Inflammatory bowel disease	MR Egger	0.467	1.722	0.457	6.486	<0.001	0.755
Amyotrophic lateral sclerosis	Inflammatory bowel disease	Weighted median	0.302	1.077	0.935	1.240		
Multiple sclerosis	Ulcerative colitis	MR Egger	0.004	1.241	1.080	1.427	<0.001	0.201
Multiple sclerosis	Ulcerative colitis	Weighted median	0.000	1.303	1.234	1.375		
Major depressive disorder	Ulcerative colitis	MR Egger	0.957	0.733	0.000	1.49E+04	<0.001	0.982
Major depressive disorder	Ulcerative colitis	Weighted median	0.309	1.337	0.764	2.342		
Bipolar disorder	Ulcerative colitis	MR Egger	0.232	0.390	0.095	1.599	<0.001	0.168
Bipolar disorder	Ulcerative colitis	Weighted median	0.231	1.140	0.920	1.411		
schizophrenia	Ulcerative colitis	MR Egger	0.471	1.173	0.768	1.791	0.394	0.755
schizophrenia	Ulcerative colitis	Weighted median	0.036	1.122	1.008	1.248		
Alzheimer's disease	Ulcerative colitis	MR Egger	0.159	1.443	0.923	2.256	0.394	0.755
Alzheimer's disease	Ulcerative colitis	Weighted median	0.388	1.081	0.906	1.290		
Parkinson's disease	Ulcerative colitis	MR Egger	0.933	1.012	0.779	1.314	0.781	0.728
Parkinson's disease	Ulcerative colitis	Weighted median	0.288	1.053	0.957	1.158		
Amyotrophic lateral sclerosis	Ulcerative colitis	MR Egger	0.404	2.720	0.332	2.23E+01	<0.001	0.581
Amyotrophic lateral sclerosis	Ulcerative colitis	Weighted median	0.525	1.056	0.892	1.250		
Multiple sclerosis	Crohn's disease	MR Egger	0.643	0.964	0.824	1.126	<0.001	0.466
Multiple sclerosis	Crohn's disease	Weighted median	0.039	0.961	0.925	0.998		
Major depressive disorder	Crohn's disease	MR Egger	0.640	50.226	0.000	6.56E+07	<0.001	0.631
Major depressive disorder	Crohn's disease	Weighted median	0.014	2.165	1.171	4.003		
Bipolar disorder	Crohn's disease	MR Egger	0.177	0.323	0.074	1.415	<0.001	0.142
Bipolar disorder	Crohn's disease	Weighted median	0.690	1.044	0.847	1.286		
Schizophrenia	Crohn's disease	MR Egger	0.973	0.992	0.638	1.543	0.226	0.738
Schizophrenia	Crohn's disease	Weighted median	0.119	1.087	0.979	1.206		
Alzheimer's disease	Crohn's disease	MR Egger	0.373	1.621	0.606	4.339	<0.001	0.393
Alzheimer's disease	Crohn's disease	Weighted median	0.045	1.189	1.004	1.408		
Parkinson's disease	Crohn's disease	MR Egger	0.666	1.060	0.824	1.362	0.852	0.997
Parkinson's disease	Crohn's disease	Weighted median	0.047	1.093	1.001	1.193		
Amyotrophic lateral sclerosis	Crohn's disease	MR Egger	0.908	1.050	0.482	2.287	<0.001	0.546
Amyotrophic lateral sclerosis	Crohn's disease	Weighted median	0.073	1.187	0.984	1.433		

*Note*: OR represents the odds ratio, or_lci95 and or_uci95 represent the lower and upper bounds of the 95% confidence interval for the OR, respectively.

Conversely, we found no evidence for causal effects of IBD (OR = 1.000, 95% CI = 0.973–1.027, *p* = 0.978), UC (OR = 0.994, 95% CI = 0.966–1.023, *p* = 0.703), or CD (OR = 1.017, 95% CI = 0.994–1.040, *p* = 0.149) on BD. Similarly, no causal relationships were observed for AD (IBD: OR = 0.988, 95% CI = 0.966–1.011, *p* = 0.313; UC: OR = 0.990, 95% CI = 0.967–1.012, *p* = 0.364; CD: OR = 0.990, 95% CI = 0.971–1.011, *p* = 0.355), PD (IBD: OR = 1.009, 95% CI = 0.967–1.052, *p* = 0.688; UC: OR = 1.007, 95% CI = 0.958–1.057, *p* = 0.796; CD: OR = 1.011, 95% CI = 0.974–1.049, *p* = 0.579), or ALS (IBD: OR = 0.995, 95% CI = 0.968–1.022, *p* = 0.714; UC: OR = 1.004, 95% CI = 0.974–1.035, *p* = 0.793; CD: OR = 1.000, 95% CI = 0.977–1.022, *p* = 0.972). Additionally, UC showed no significant causal effect on SCZ (OR = 1.010, 95% CI = 0.981–1.040, *p* = 0.508). All reported associations had *p*‐values exceeding the threshold for statistical significance (*p* > 0.05), with effect estimates consistently approximating the null value.

### 3.2 Reverse Analysis

**FIGURE 3:**
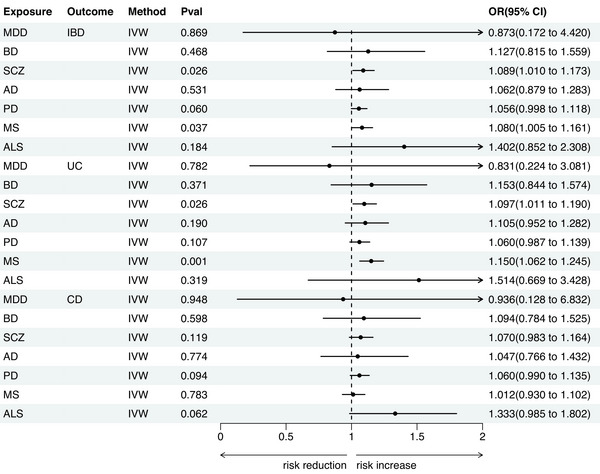
MR estimates of seven major neuropsychiatric disorders on IBD. **Abbreviations**: AD, Alzheimer's disease; ALS, amyotrophic lateral sclerosis; BD, bipolar disorder; CD, Crohn's disease; IBD, inflammatory bowel disease; MDD, major depressive disorder; MR, Mendelian randomization; MS, multiple sclerosis; PD, Parkinson's disease; SCZ, schizophrenia; UC, ulcerative colitis.

In the reverse analysis, we used the IVW method to identify the causal effects of SCZ and MS on IBD and UC (as shown in Figure 3, Table 1). Specifically, gene proxies for SCZ were associated with an increased risk of IBD (OR = 1.089, 95% CI = 1.010–1.173, *p* = 0.026) and UC (OR = 1.097, 95% CI = 1.011–1.190, *p* = 0.026). Gene proxies for MS were associated with an increased risk of IBD (OR = 1.080, 95% CI = 1.005–1.161, *p* = 0.037) and UC (OR = 1.150, 95% CI = 1.062–1.245, *p* = 0.001).

Conversely, we found no evidence for causal effects of MDD (IBD: OR = 0.873, 95% CI = 0.172–4.420, *p* = 0.869; UC: OR = 0.831, 95% CI = 0.224–3.081, *p* = 0.782), BD (IBD: OR = 1.127, 95% CI = 0.815–1.559, *p* = 0.468; UC: OR = 1.153, 95% CI = 0.844–1.574, *p* = 0.371), AD (IBD: OR = 1.062, 95% CI = 0.879–1.283, *p* = 0.531; UC: OR = 1.105, 95% CI = 0.952–1.282, *p* = 0.190), PD (IBD: OR = 1.056, 95% CI = 0.998–1.118, *p* = 0.060; UC: OR = 1.060, 95% CI = 0.987–1.139, *p* = 0.107), or ALS (IBD: OR = 1.402, 95% CI = 0.852–2.308, *p* = 0.184; UC: OR = 1.514, 95% CI = 0.669–3.428, *p* = 0.319) on IBD or UC. Similarly, none of the seven neuropsychiatric disorders showed significant causal effects on CD (MDD: OR = 0.936, 95% CI = 0.128–6.832, *p* = 0.948; BD: OR = 1.094, 95% CI = 0.784–1.525, *p* = 0.598; SCZ: OR = 1.070, 95% CI = 0.983–1.164, *p* = 0.119; AD: OR = 1.047, 95% CI = 0.766–1.432, *p* = 0.774; PD: OR = 1.060, 95% CI = 0.990–1.135, *p* = 0.094; ALS: OR = 1.333, 95% CI = 0.985–1.802, *p* = 0.062), with all *p*‐values exceeding the threshold for statistical significance (*p* > 0.05).

### 3.3 Sensitivity Analysis

As shown in Table 1, the Cochran *Q* test indicated that most MR estimates exhibited heterogeneity (*p* < 0.05). However, this is permissible within our chosen random‐effects IVW model. We observed non‐zero intercepts in four Egger intercept tests (IBD for BP, UC for ALS, CD for BP, and CD for ALS), with significant results, indicating the presence of horizontal pleiotropy (*p* < 0.05). Therefore, the MR‐PRESSO method was employed to identify potential outliers in these four estimates. Ultimately, no bias due to outliers was detected in the re‐evaluated MR estimates, further confirming the robustness of our results (IBD for BP: *p* = 0.723; UC for ALS: *p* = 0.645; CD for BP: *p* = 0.137; CD for ALS: *p* = 0.656).

## 4 Discussion

In this study, we utilized MR methods to investigate the causal relationships between IBD and seven major neuropsychiatric disorders. Our analysis revealed that IBD and CD have positive causal effects on MDD and SCZ, and similarly, SCZ is positively correlated with an increased risk of IBD and UC. There is a bidirectional positive association between IBD, UC, and MS, while CD has a positive causal effect on MS. In contrast, we found no evidence supporting the causal effects of MDD, BD, AD, PD, or ALS on IBD or UC. Sensitivity analyses further reinforced the robustness of the MR estimates. These results provide novel insights into the complex interplay between gastrointestinal and neuropsychiatric conditions, highlighting the potential role of inflammatory processes in the pathogenesis of both. The association between IBD and depression has been extensively documented (Bisgaard et al. 2022). The prevalence of depressive symptoms in individuals with IBD ranges from 21% to 25.2% (Barberio et al. 2021, Mikocka‐Walus et al. 2016). Studies have shown that depression is a significant comorbidity of IBD, with a cohort study revealing an increased risk of new‐onset depression, particularly around ten years following an IBD diagnosis (Bisgaard et al. 2023). This highlights the chronic and long‐term impact that IBD can have on mental health. Our analysis further supports this relationship, indicating that both IBD and CD may elevate the risk of developing depression. However, no evidence was found to support a causal relationship in the reverse direction, suggesting that depression may not significantly contribute to the onset of IBD. These findings underscore the need for clinicians to monitor and address mental health in IBD patients, especially those with a long history of the disease.

In the current MR estimates, IBD is associated with an increased risk of SCZ, and vice versa; however, the association between CD, UC, and SCZ is inconsistent. Previous cohort studies have demonstrated a significant association between SCZ and an increased risk of subsequent IBD, although this was conducted in an East Asian population (Sung et al. 2022). A European cohort study involving 7,704 participants found that individuals with a history of any autoimmune disease had a 45% increased risk of developing SCZ, with a higher prevalence of SCZ among patients compared to controls (Eaton et al. 2006). This suggests a bidirectional risk between SCZ and autoimmune diseases, which was not observed in our study. Additionally, the relationship between BD and IBD remains perplexing. Research by Kao et al. indicated that patients with IBD are more likely to develop BD (Kao et al. 2019). However, a large cohort study based on a Danish population confirmed no association between IBD and BD, either before or after the diagnosis (Bisgaard et al. 2023), which is consistent with the findings of our MR analysis.

Neurological disorders demonstrate significantly higher prevalence among IBD patients. Accumulating evidence substantiates elevated rates of headache disorders, migraine, and myasthenia gravis in this population (Leitão et al. 2021, Leitão et al. 2025, Gondim Fde et al. 2014, Rocha et al. 2017). A prior comparative study revealed substantially greater incidence of neuromuscular disorders in both CD and UC patients relative to those with gastritis or dyspepsia (Gondim Fde et al. 2015). Furthermore, emerging data implicate peripheral neuropathy as a clinically relevant extraintestinal manifestation of IBD (Gondim Fde et al. 2015, Gondim et al. 2005). Regarding neurological disorders, we identified a bidirectional association between IBD and MS. Notably, causal relationships were also confirmed between UC, CD, and MS. A meta‐analysis by Kosmidou et al. investigated the correlation between MS and IBD (Kosmidou et al. 2017). The study found that individuals with either IBD or MS exhibited a 50% increased risk of developing comorbidities associated with MS or IBD, respectively. However, this finding was not replicated in patients with CD or UC (Kosmidou et al. 2017). Additionally, several previous studies suggested that IBD may increase the risk of PD (Weimers et al. 2019, Villumsen et al. 2019), while others found IBD, CD, and UC to be associated with a lower risk of PD (Camacho‐Soto et al. 2018). Growing evidence highlights the significant role of inflammation in AD (Chen and Holtzman 2022). Research by Aggarwal et al. supports the notion that IBD could be an independent risk factor for AD (Aggarwal et al. 2023). Another study found that IBD patients have a higher risk of developing dementia within 15 years compared to non‐IBD patients (Zingel et al. 2021). However, similar to previous MR analyses (Zeng et al. 2023), we did not observe an association between IBD and PD or AD. Unlike earlier MR analyses (Li et al. 2021), our study does not support a positive association between IBD and ALS. Currently, there is a lack of observational evidence regarding the association between IBD and ALS, and further research is warranted. Notably, our study utilized larger summary datasets, providing new genetic‐level evidence for the causal relationships between IBD and neurodegenerative diseases.

Overall, our study underscores the association between IBD and its clinical subtypes with neuropsychiatric disorders. The current findings diverge from some observational evidence, potentially due to design flaws such as confounding factors in previous studies. Our MR analysis typically reflects lifetime effects, which may result in discrepancies compared to the short‐term follow‐up periods of cohort studies.

This study has several advantages. Firstly, we systematically assessed the potential causal associations between IBD and its subtypes with seven major neuropsychiatric disorders, enhancing the understanding of both extra‐intestinal manifestations of IBD and the intestinal manifestations of neuropsychiatric disorders. Notably, the use of MR methods effectively reduced confounding factors and reverse causation interference. Additionally, sensitivity analyses reinforced the robustness of our MR analysis. However, our study has some limitations. Firstly, our results are limited to European populations, and the findings may not apply to other populations. Second, since the GWAS summary statistics utilized in this study lacked individual‐level sex‐stratified data (which were not disclosed in the original studies), we were unable to conduct sex‐specific stratification analyses or investigate potential nonlinear causal relationships. Third, current evidence remains insufficient to comprehensively elucidate the genetic effects on both IBD subtypes and neuropsychiatric disorders, necessitating further investigation to unravel this complex interplay. Thus, further research is needed to validate our results.

## 5 Conclusion

In summary, our results support the potential causal effects between IBD and its subtypes and neuropsychiatric disorders. These findings provide genetic evidence further highlighting the interaction between the gut‐brain axis. Clinicians should take this into account to develop appropriate clinical management strategies. Further research is needed to validate our findings.

## Author Contributions

**Jiawei Lei**: writing – original draft, writing – review and editing, methodology, formal analysis, validation, visualization, data curation, software, and investigation. **Liping Gong**: writing – review and editing, visualization, and methodology. **Shan Wei**: methodology, visualization, writing – review and editing. **Junshan Deng**: conceptualization, writing – original draft, writing – review and editing, supervision, and resources.

## Funding

The authors have nothing to report.

## Ethics Statement

All data used in this study were made publicly available in the original GWAS study, and appropriate patient consent and ethical approval were obtained.

## Conflicts of Interest

The authors declare no conflicts of interest.

## Data Availability

Data supporting the findings of this study are available from the article/Supplementary material.
